# The multimodal treatment in headaches

**DOI:** 10.1186/1129-2377-16-S1-A47

**Published:** 2015-09-28

**Authors:** Marco R Lacerenza, Fabia Schoss, Licia Grazzi

**Affiliations:** Casa di Cura S.Pio X, “Opera San Camillo” Foundation, Milan, Italy; Milan, Italy; Headache Centre, Neurological Institute C. Besta IRCCS Foundation, Milan, Italy

Primary headaches are common debilitating disorders with high prevalence and significant socioeconomic and personal impacts. Idiopathic headaches affect all aspects of the individual's life and are the result of complex interaction of biological, psychological, and environmental factors. In patients with chronic headaches the efficacy of pharmacological treatments is often not satisfactory. Pain and disability can potentially induce an escalation of analgesics/triptans intake leading to medication-overuse headache [1]. Experiencing pain can trigger a cascade of neurological events that lead to an altered psychological state and therefore to aberrant behaviors. Moreover, prior psychological states and psychiatric comorbidities can confer a heightened risk for pain chronicity due to processes such as cross sensitization, where exposure to stress in the past results in greater sensitivity to other seemingly unrelated stimuli[2]. Accordingly, the processes of sensitization in headache patients, can be expressed both in the peripheral and central nervous systems, contributing to pain chronicization. Given the multidimensional nature of chronic pain, efficacious assessment and treatment requires a comprehensive, multiaxial approach considering every aspect of the individual's life[3, 4]. Modification of lifestyle habits could play a role in preventive strategies of primary headaches, especially in childhood and in adolescence[5]. The non-pharmacological therapies can be part of a multimodal treatment or an alternative therapy in the case of pregnancy, breast feeding, multiple therapies for comorbid diseases, poor tolerability of drugs, childhood and elderly[6]. Acupuncture and biofeedback are considered the first-choice for the prophylaxis of tension-type headache[6]. Many other non-pharmacological treatments are useful in the prevention of primary headaches, although further well-conducted studies are needed to support their efficacy. They include physiotherapy/physical exercise, progressive muscle relaxation training, short-term psychotherapy and cognitive-behavioural therapy[6]. Another promising intervention is Mindfulness meditation, which is characterized by deliberately focusing on the present moment in a non-judgmental way[7]. Several recent neuroimaging studies suggest that meditation may modulate pain through several mechanisms[8]. It may reduce the saliency of noxious stimuli through attentional focusing, and promote pain modulation reducing expectations of impending noxious stimuli. Moreover, it could induce beliefs related to the promotion of pain relief and refraining from catastrophic thinking. The headache patient can be difficult to manage. We propose the setting of a multimodal treatment, shared by the patient who has to be considered an integral part of care, aimed at improving all aspects of the individual's life.
